# Effects of Flavonoids in *Fructus Aurantii Immaturus* on Carcass Traits, Meat Quality and Antioxidant Capacity in Finishing Pigs

**DOI:** 10.3390/antiox13111385

**Published:** 2024-11-13

**Authors:** Zekun Yang, Qiuping Guo, Xiangfeng Kong, Yixing Li, Fengna Li

**Affiliations:** 1 Guangxi Key Laboratory of Animal Breeding, Disease Control and Prevention, College of Animal Science and Technology, Guangxi University, Nanning 530004, China; 2218391053@st.gxu.edu.cn; 2 Key Laboratory of Agro-Ecological Processes in Subtropical Region, Institute of Subtropical Agriculture, Chinese Academy of Sciences, Changsha 410125, China; guoqiuping@isa.ac.cn (Q.G.); nnkxf@isa.ac.cn (X.K.); 3 College of Modern Agricultural Sciences, University of Chinese Academy of Sciences, Beijing 100049, China

**Keywords:** carcass traits, meat quality, antioxidant capacity, FFAI, finishing pig

## Abstract

This experiment aimed to explore the effects of flavonoids in *Fructus Aurantii Immaturus* (FFAI) on carcass traits, meat quality, and the antioxidant capacity of finishing pigs. The results indicated that the addition of an appropriate amount of FFAI into their diet could significantly reduce the backfat thickness and perirenal fat percentage of finishing pigs, as well as the drip loss, water-holding capacity, shear force, and the levels of lactate, glucose-6-phosphate, glucose, ATP, phosphofructokinase, and pyruvate in the longissimus dorsi (LD) muscle. It also elevated the levels of flavor amino acids such as glutamate, serine, and threonine, and enriched the composition of flavor substances, including benzene and octanal, which significantly contributed to the enhancement of pork flavor. Furthermore, it enhanced the expression levels of *MyHC I* and *MyHC IIa*. In summary, the appropriate addition of FFAI to the diet could improve the carcass traits, meat quality, and antioxidant capacity of finishing pigs. The optimal level of FFAI supplementation is 0.12%.

## 1. Introduction

As society progresses and living standards rise, individuals are placing increasing emphasis on health and the quality of their food [1], with a particular emphasis on the nutritional benefits of meat products and their ramifications for wellbeing [2]. Pork is a widely eaten meat, which is an important source of protein which affects human health [3]. In recent years, more and more reports have shown that the incorporation of feed additives, including vitamins [4,5], amino acids [6,7], and plant extracts [8], can improve the quality of pork, including color, water-holding capacity, amino acid composition, flavor, antioxidant capacity, and muscle fiber characteristics. The amino acid composition and flavor compounds present in muscle tissues are recognized as crucial indicators of meat flavor [9,10]. Currently, the detection methodologies for volatile organic compounds encompass electronic noses and gas chromatography-mass spectrometry (GC-MS) [11]. In addition, the characteristics of muscle fibers and antioxidant capacity are intimately associated with various facets of meat quality attributes, including water-holding capacity, meat color, pH value, and tenderness [12,13]. However, due to increased consumer health awareness and concerns about food safety, the current trend is to minimize the use of synthetic materials [14]. Therefore, researchers are interested in finding natural feed additives to improve pork quality [15].

*Fructus Aurantii Immaturus* is a traditional Chinese herbal medicine, and its fruits and seeds are widely used to treat various diseases. It contains a variety of biologically active ingredients. Among them, the FFAI has been proven to have the effects of promoting digestion, antibacterial, anti-inflammatory, and anti-oxidation [16]. Flavonoids are natural phenolic compounds [17], which are widely present in various parts of plants and have a diverse array of health-promoting effects, especially antioxidant properties [18,19,20]. For example, plant flavonoids may improve intestinal homeostasis by modulating the structure of intestinal microbial populations to increase levels of lactobacilli and bifidobacterial [21]. It has also been shown that plant flavonoids have potential use as natural feed additives, replacing antibiotics and enhancing antioxidant capacity, immune function, growth performance, as well as intestinal health in farm animals [22]. Buckwheat flavonoids improve growth performance, antioxidant ability, and nutrient digestibility of piglets in pig breeding [23,24]. *Eucommia ulmoides* flavonoids (flavonoids extracted from leaves by shade drying and fine powder treatment) can alleviate the growth inhibition and liver and spleen lesions of weaned piglets induced by DON, and improve the body’s antioxidant capacity and anti-inflammatory ability [25]. It has been shown that FFAI can inhibit the inflammatory response by downregulating the overexpression of colonic inflammatory cytokines [26], improve the intestinal microflora, and enhance the antioxidant properties in mice with colitis [27]. Additional research has demonstrated that FFAI possesses the ability to inhibit lipid accumulation in adipocytes, thereby contributing to the reduction in obesity [28]. However, to date, the impact of FFAI on the quality of finishing pork remains unreported, and the present study aims to address this gap in the literature.

Therefore, the aim of this study was to assess the impact of supplementing the diet with FFAI on carcass traits, meat quality, and antioxidant capacity in finishing pigs. This study offers novel evidence supporting the use of FFAI as a beneficial feed additive for improving the taste, flavor, and nutritional quality of pork.

## 2. Materials and Methods

### 2.1. Ethics Statement

In this study, the animal study protocol was approved by the Animal Ethics Committee of the Institute of Subtropical Agriculture at the Chinese Academy of Sciences (Protocol No. ISA-2024-0094).

### 2.2. Animals and Diets

The castrated Duroc × Landrace × Yorkshire (DLY) hybrid boars from the same batch and possessing an initial body weight of 70 kg were selected as the research subjects. The experimental pigs were randomly assigned to three groups using a single-factor experimental design, with each group containing 10 replicates and each replicate comprising 6 pigs. The formal experiment was divided into two stages: the early stage of fattening (70–90 kg) and the late stage of fattening (90–125 kg). In these two stages, the three groups received different levels of FFAI supplementation in the concentrate portion of the basal diet: 0, 0.12%, and 0.24%. FFAI was extracted from dried citrus fruit by using a fine powder treatment. It was synthesized by Zhangjiajie Jiyuan Technology Co., Ltd., Zhangjiajie, China. The composition of the FFAI was detailed in the Supplementary Materials (Table S1). The feeding and management of the experimental pigs in each group were conducted in accordance with the regulations of the experimental pig farm’s feeding and management, with free access to drinking water and feeding. The feeding and management of each treatment group were consistent. During the feeding period, weekly records were maintained for both feed intake and live weight of the pigs. The pre-feeding time was 7 days. When the pigs reached an average body weight of about 125 kg, the growth test was completed for 56 days. Subsequently, a total of 30 pigs, with 10 pigs randomly chosen from each group, were assessed for carcass traits and meat quality-related indicators following slaughter. The corn-soybean meal basal diet was used in the experiment, which was prepared according to the nutritional level recommendations of NRC (2012). The composition and nutrient levels of the basal diet were provided in the Supplementary Materials (Table S2).

### 2.3. Carcass Traits and Sample Collection

Prior to slaughter, all pigs underwent a 12 h fasting period. Each pig was subjected to slaughter through electric shock, followed by bloodletting, hair removal, peeling, viscera removal, and an incision made from the midline. The pigs were then slaughtered, and samples were obtained at the local slaughterhouse. Then, blood was drawn from the jugular veins of the pigs and were allowed to stand for 0.5 h, and then the blood samples were centrifuged (3000× *g*, 4 °C, 15 min) to separate the serum. Carcass and perirenal fat (fat around the kidneys) weights were recorded, and carcass straight lengths and oblique lengths were measured. Measurements and records of the backfat thickness at the shoulder’s maximum thickness (approximately between the 4th to 5th ribs), at the last rib, and at the last lumbar vertebrae were conducted to determine the average backfat thickness. The loin eye area was measured at the level situated between the 6th and 7th rib. Two pieces of 50 g LD muscle samples were harvested from the left side of the carcass, positioned between the 5th and 8th rib, put into the sample bag and put into the refrigerator, and then transferred to the −20 °C refrigerator for storage, for glycolysis related indicators; the cooking loss, drip loss, pH value (45 min and 24 h), tasting experiment, meat color, and shear force of the LD muscle at the 10th–16th rib and thoracolumbar junctions were measured. The LD muscle between the 8th and 10th rib was wrapped in aluminum foil paper and sub-packed in a 2 mL cryopreservation tube. It was first stored in a liquid nitrogen tank and then transferred to a refrigerator, maintained at −80 °C to be tested for various indicators. In addition, the LD muscle sample was snap-frozen in liquid nitrogen and preserved at −80 °C for future molecular biological assessments.

### 2.4. Determination of Meat Quality

Following the calibration using the buffers of pH 4.6 and 7.0, the pH value of the LD muscle was determined using a pH meter (Matthaus pH star, Lenzkirch, Germany). Additionally, the a*, b*, and L* values of the LD muscle, located between the 10th and 12th rib, were assessed using a colorimeter (CR-410, Konica Minolta Sensing Inc., Osaka, Japan) at 45 min and 24 h post-slaughter. The water loss percentage was measured using a meat press (Bulader-M10, Beijing Brad Technology Development Co., Ltd., Beijing, China). Approximately 100 g of LD muscle at the 12th–14th rib was taken, and the muscle was trimmed into 1 cm × 1 cm × 3 cm meat pieces parallel to the muscle fibers. The meat sample was lifted with a metal hook and wrapped with a fresh-keeping bag to avoid contact between the meat and the fresh-keeping bag. The meat sample was hung at 4 °C for a period of 24 h, and the weight variation between the front and back of the meat sample was documented [29]. The muscle sample had a weight of approximately 120 g, was placed in a steaming cage, and was subjected to steaming in boiling water for 30 min. Subsequently, the meat sample was allowed to cool for 30 min, followed by drying with filter paper. The difference in weight before and after the drying process was then calculated. Muscle samples, each weighing approximately 250 g, were heated in water at 80 °C until they reached a core temperature of 75 °C, then cooled down to room temperature. From each sample, eight cylindrical cores, each measuring 1.27 cm in diameter, were excised parallel to the muscle fibers. The shear force was assessed using a texture analyzer (TMS-pro, TFC, Sterling, Virginia, USA), with measurements taken perpendicular to the muscle fibers. The meat samples were then kept at 4 °C for a period of 24 h. Marbling and meat color scores were assessed by three experienced evaluators using the criteria of the National Pork Producers Committee (NPPC) [30]. The LD muscle was cut into 1 cm^3^ cubes, boiled in boiling water for 5 min, and cooled to room temperature for tasting. There were 12 tasters (Experienced and professionally trained personnel). Due to individual differences, in order to eliminate inaccurate sensory evaluation, 2 samples from the same meat were provided to each taster, and the tasters were blinded to the origin of the samples beforehand. The description and definition of elasticity, juiciness, chewiness, tenderness, appearance, aroma, and taste of meat samples are shown in Table S3. Using 0–10 grade score. 0 points means very poor, 5 points means general, 10 points means excellent.

### 2.5. Electronic Nose

Each sample from the LD muscle groups was boiled in boiling water to reach a central temperature of 70 degrees Celsius. In the sample bottle, 2 g of the muscle sample was weighed, and the cap of the bottle was tightened. After standing for half an hour, an electronic nose probe was inserted into the bottle, and analysis of each sample was collected for 100 s. The changes in odor characteristics among the three groups of LD muscles were evaluated using an electronic nose (iNose, Isenso, Ruifen Trading Co., Ltd., Shanghai, China), with odor changes being distinguished using a selective sensor array. The performance description of each sensor is provided in the Supplementary Material (Table S4).

### 2.6. SPME-GC-MS Detection of Volatile Flavor Substances

The LD muscle samples were thawed and removed from the superficial connective tissue. 2g of the sample was weighed into an extraction vial and then mixed well. Following this, 2 µL of the internal standard (o-chlorophenylphenol, 200 µL/mL) was added and placed in a septum on the vial and tightly sealed. It was then stabilized in a 70 °C water bath for 30 min, before it was inserted in the aged extraction head, where it was extracted for 30 min at 70 °C using headspace extraction. After the extraction was complete, the extraction head was removed and inserted into the GC-MS sample inlet. It was then kept inside for 5 min before it was removed. Instrument parameters: QP2010 gas chromatography-mass spectrometry system; chromatographic column Rtx-5MS (30 m × 0.25 mm × 0.25 μm); injection port was maintained at 250 °C, with a resolution time of 4 min and no split; the carrier gas was hydrogen (99.999%) with a flow rate of 1.78 mL/min; the temperature program: hold at 40 °C for 3 min, then increase to 70 °C at a rate of 4 °C/min with no hold, followed by an increase to 230 °C at a rate of 3 °C/min and hold for 5 min, with a total program time of 69 min; the interface temperature was 240 °C; the ion source temperature was 200 °C; ionization mode: EI+, 70 eV; scanning mode was full scan. Based on the total ion current chromatogram, compounds were identified by searching the Nist and Flavor DB libraries and analyzed using the internal standard normalization method for the detected compounds.

### 2.7. Determination of Antioxidant Capacity and Glycolysis Index

The tissue samples were homogenized using a 10% PBS solution in a weight-to-volume ratio of 1:9, followed by centrifugation to isolate the supernatant. An ELISA assay was then conducted according to the established protocol [31]. The antioxidant and glycolysis indexes of the serum and LD muscle samples were determined by the kit of the Nanjing Jiancheng Biological Research Institute. Sample addition: the blank hole, standard hole, and sample hole to be tested were set respectively; accurately pipetting 50 μL of the standard sample onto the enzyme-labeled coating plate; adding 40 μL of sample diluent to the sample hole, followed by the addition of 10 μL of sample to achieve a final dilution of 5 times. The plate was then sealed with a sealing film and incubated at 37 °C for 30 min. For the re-dispensing liquid, the concentrated washing liquid was diluted 30 times with distilled water for subsequent use. After another wash, 50 μL of enzyme-labeled reagent was added to each well, excluding the blank well, and the plate was incubated again. Following this, 50 μL of the color of reagent A and then 50 μL of the color of reagent B were added to each well. The mixture was gently shaken, and the plate was incubated at 37 °C for 10 min. The reaction was terminated by adding 50 μL of the termination solution to each hole. Finally, the blank wells were adjusted to zero, and the OD values of each well were sequentially detected at a wavelength of 450 nm using a multifunctional microplate reader, SpectraMax i3X A1705 (Molecular Devices, LLC, San Jose, CA, USA). The determination should be completed within 15 min after adding the termination solution. Antioxidant indicators included superoxide dismutase (SOD), glutathione peroxidase (GSH-Px), catalase (CAT), total antioxidant capacity (T-AOC) activity, and malondialdehyde (MDA) level. Glycolysis indicators included lactic acid (Lac), glucose-6-phosphate (G6P), glucose (Glu), glycogen (Gly), adenosine triphosphate (ATP), phosphofructokinase (PFK-1), creatine kinase (CK), lactate dehydrogenase (LDH) and pyruvate (Pyr). All procedures were followed according to the manufacturer’s guidelines [13].

### 2.8. Determination of Flavor Amino Acids

Flavor amino acid composition analysis was conducted according to the method previously described [32,33]. Approximately 0.1 g of freeze-dried meat powder was homogenized in 5 mL 0.01 M hydrochloric acid. The mixture was then centrifuged at 5000× *g* rpm for 5 min, 0.5 mL of supernatant was taken, and 0.5 mL of 8% yellow-based salicylic acid was added. After allowing the mixture to sit overnight, it was centrifuged at 12,000× *g* rpm revolutions per minute for 10 min. The supernatant was then decanted, and the process was repeated with another centrifugation at 12,000× *g* rpm for 10 min. Through the membrane (0.45 μm or 0.22 μm) and subsequently analyzed for the content of flavor amino acids using an L8900 amino acid analyzer (L8900, Hitachi, Tokyo, Japan). All procedures were followed according to the manufacturer’s guidelines [13]. Chromatographic conditions: chromatographic column: AccQ-Tag Ultra C18 column (1.7 μm, 2.1 mm × 100 mm); mobile phase A: AccQ Tag Eluent A; mobile phase B: 10% AccQ Tag Eluent B; mobile phase C: water; mobile phase D: AccQ Tag Eluent B. Column temperature: 43 °C; flow rate: 0.7 mL/min; detection wavelength: 260 nm; injection volume: 1 μL. The gradient elution procedure is shown in Table S5.

### 2.9. Real-Time Quantitative PCR

Total RNA was isolated from the LD muscle samples utilizing TRIzol reagent (Invitrogen, Carlsbad, CA, USA), and its concentration and purity were determined using an ultraviolet spectrophotometer to ensure that the absorbance ratio (OD260/280) was between 1.8 and 2.2. After standardizing the RNA concentration to 1 μg/μL, cDNA synthesis was performed using the EvoM-MLV reverse transcription kit (Hunan Biological Engineering Co., Ltd., Jinshi, Hunan, China). Real-time quantitative PCR analysis was conducted using the SYBR Green Premix Ex Taq HS qPCR kit (Hunan Biological Engineering Co., Ltd., Jinshi, Hunan, China). The reaction mixture comprised 5 μL of the SYBR Green Premix Ex Taq HS, 4.2 μL of cDNA template, and 0.4 μL of primer pairs, amounting to a total volume of 10 μL. The PCR protocol was initiated with a pre-denaturation step at 95 °C for 30 s, followed by 40 cycles of denaturation at 95 °C for 5 s and extension at 60 °C for 30 s. The expression levels of myosin in the muscle fibers, categorized as slow oxidative (type I), fast oxidative (type IIa), intermediate (type IIx), and fast glycolytic (type IIb), were determined using *β-actin* as an internal reference gene. The mRNA levels of the genes were quantified using the 2^−ΔΔCt^ method. The primer sequences utilized are listed in Table S6.

### 2.10. Statistical Analysis

The experimental data were initially organized using Excel 2016, and SPSS 22.0 software was used to perform one-way ANOVA to assess the variance between groups. For multiple comparisons, Tukey’s post hoc test was employed. The data are presented as mean ± SEM. Various analyses, including principal component analysis, flavor difference analysis, and Mantel tests for electronic nose data, were conducted using the ggpolt2, pheatmap, and linkET packages in R version 4.3.1. Statistical significance was determined at *p* < 0.05.

## 3. Result

### 3.1. Growth Performance and Carcass Characteristics

As shown in Table 1, dietary FFAI supplementation did not significantly affect the growth performance indicators of finishing pigs. However, the final body weight and average daily gain in the FFAI supplementation group were slightly higher than in the control group, and the F/G in the FFAI supplementation group was slightly lower than in the control group.

As shown in Table 2, compared with the control group, the addition of 0.12% FFAI to the diet significantly reduced backfat thickness and perirenal fat percentage (*p* < 0.05). In addition, the addition of FFAI to the diet could increase the slaughter rate and loin eye area to a certain extent, but it is not significant compared with the control group.

### 3.2. Meat Quality

As shown in Table 3, compared with the control group, the addition of 0.12% FFAI to the diet significantly increased the redness values of the pork at 45 min and 24 h, as well as the meat color scores. Additionally, it significantly reduced the drip loss and shear force of the pork (*p* < 0.05). Furthermore, the addition of 0.24% FFAI was able to significantly reduce the water loss and drip loss of the pork (*p* < 0.05).

As shown in Table 4, the FFAI supplementation group showed significant improvement in tenderness, appearance, aroma, and flavor compared with the control group (*p* < 0.05).

### 3.3. Flavor Analysis of LD Muscle

As presented in Table 5, when compared to the control group, the contents of valine and isoleucine in bitter amino acids, lysine in other amino acids, and glutamic acid in umami amino acids, were significantly increased in the diet supplemented with 0.24 % FFAI (*p* < 0.05). When compared with the other two groups, the contents of serine and threonine in sweet amino acids were significantly increased in the diet supplemented with 0.12 % FFAI group (*p* < 0.05).

**Figure 1:**
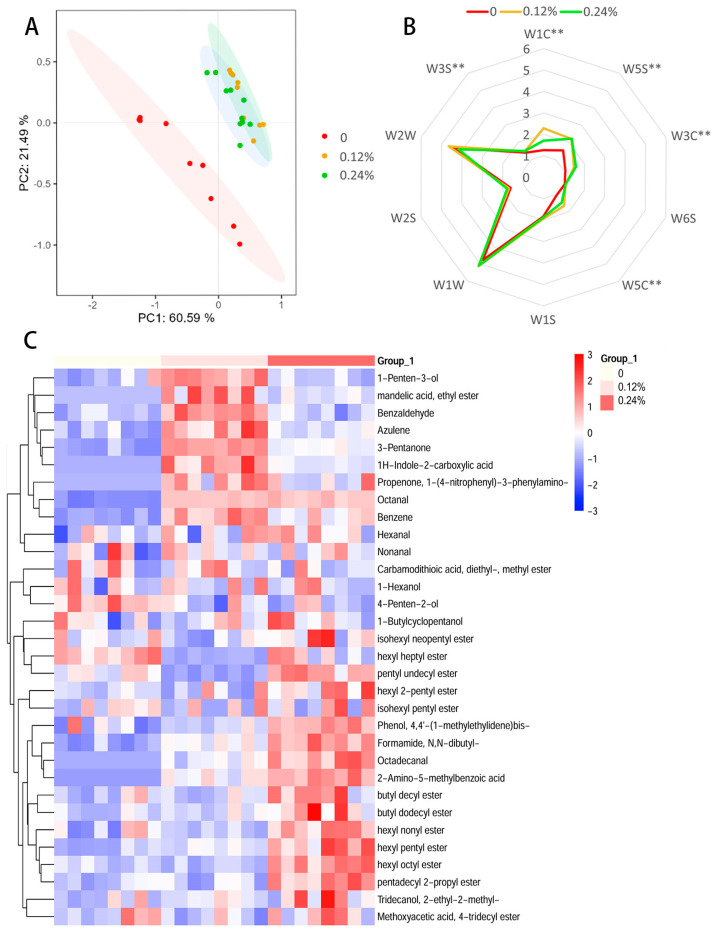
Analysis of flavor substances in LD muscle. (**A**) Electronic nose PCA diagram; (**B**) radar diagram of odor response values; (**C**) Cluster heat map of differential flavor substances in LD muscle. Values are the mean ± SEM (*n* = 8), Significance was expressed as ** *p* < 0.01.

Figure 1A illustrates the results of the principal component analysis (PCA) conducted on the response values of the electronic nose for three groups. Two principal components, PC1 and PC2, were identified, with the first principal component contributing 60.59% and the second contributing 21.49% to the variance. The combined contribution rate of the first two principal components is 82.08%, and the total contribution rate exceeds 80%, indicating that the results can fully reflect the overall characteristics of the three groups and effectively differentiate between them. Notably, there is a significant difference in the volatile components between the control group and the experimental groups, while the experimental groups show some similarity in their volatile components. In the radar chart (Figure 1B), the response values of the LD muscle in the group with added FFAI in the feed show significant differences compared with the control group in terms of sensitivity to aromatic compounds (W1C), nitrogen oxides (W5S), benzene (W3C), alkane aromatic compounds (W5C), and alkanes (W3S) (*p* < 0.01).

As can be seen from Figure 1C, the flavor substances in the LD muscle of the three groups were clustered and analyzed. The results showed that there were significant differences in the flavor substances among the three groups (Figure 1C). Compared with the control group, dietary supplementation with FFAI significantly increased the contents of 3-pentanone and octanal in flavor substances (*p* < 0.05). Dietary 0.12% FFAI supplementation also significantly increased the contents of 1-penten-3-ol, benzene, and benzaldehyde in flavor substances (*p* < 0.05). The above flavor substances had sweet (benzene) and citrus flavor (octanal) odors. It may be the characteristic substance of pork flavor improvement in the FFAI group.

### 3.4. Antioxidant Capacity

**Figure 2:**
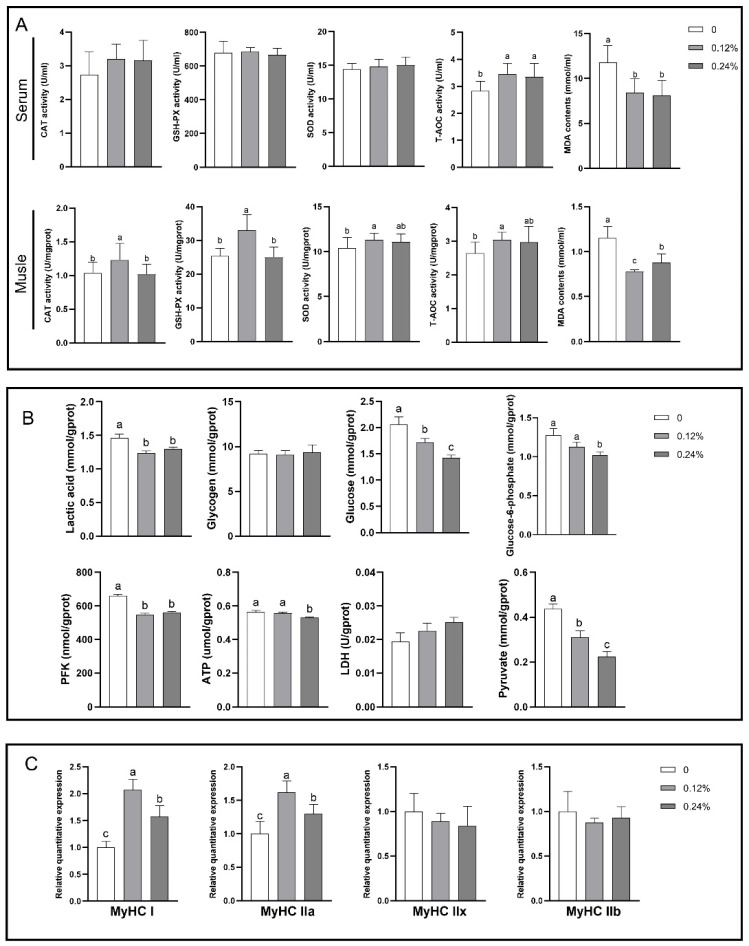
Effects of dietary FFAI supplementation on the antioxidant capacity in serum and LD muscle (**A**), glycolytic potential (**B**), and muscle fiber types in LD muscle (**C**) of finishing pigs. CAT, catalase; T-AOC, total antioxidant capacity; GSH-Px, glutathione peroxidase; SOD, superoxide Dismutase; MDA, malondialdehyde, ATP, Adenosine Triphosphate; PFK, Phosphofructokinase; LDH, Lactate Dehydrogenase. Different groups with different lower case letters above the bar graphs indicate significant differences between groups (*p* < 0.05). Values are the mean ± SEM (*n* = 8).

As shown in Figure 2A, in the serum, the T-AOC activity was notably elevated in the group receiving FFAI supplementation, compared to the control group (*p* < 0.05). Conversely, the MDA content was markedly decreased in the FFAI-supplemented group, relative to the control group (*p* < 0.05). In the LD muscle, the activities of CAT and GSH-PX were significantly higher in the 0.12% FFAI group compared to the other two groups (*p* < 0.05). Additionally, the activities of SOD and T-AOC were also notably elevated in the 0.12% FFAI group when compared to the control group (*p* < 0.05). The MDA content was significantly lower in the FFAI group compared to the control group (*p* < 0.05). Furthermore, the MDA content in the 0.12% FFAI group was notably lower than that in the other two groups (*p* < 0.05).

### 3.5. Glycolytic Potential and Gene Expression in LD Muscle

As illustrated in Figure 2B, dietary supplementation with 0.12% FFAI significantly decreased the concentrations of lactic acid, glucose, PFK, and pyruvate in the LD muscle, compared to the control group (*p* < 0.05). Dietary supplementation of 0.24% FFAI significantly reduced the contents of lactic acid, glucose-6-phosphate, glucose, ATP, phosphofructokinase, and pyruvate in LD muscle (*p* < 0.05).

As shown in Figure 2C, dietary supplementation with FFAI significantly increases the expression levels of *MyHC I* and *MyHC IIa* compared to the control group (*p* < 0.05). Moreover, the expression levels of *MyHC I* and *MyHC IIa* were significantly higher in the 0.12% FFAI supplementation group compared to the other two groups (*p* < 0.05).

### 3.6. Correlation Analysis Between Antioxidant Capacity and Meat Quality, Glycolysis and Muscle Fiber Type in Finishing Pigs Based on Mantel-Test

**Figure 3:**
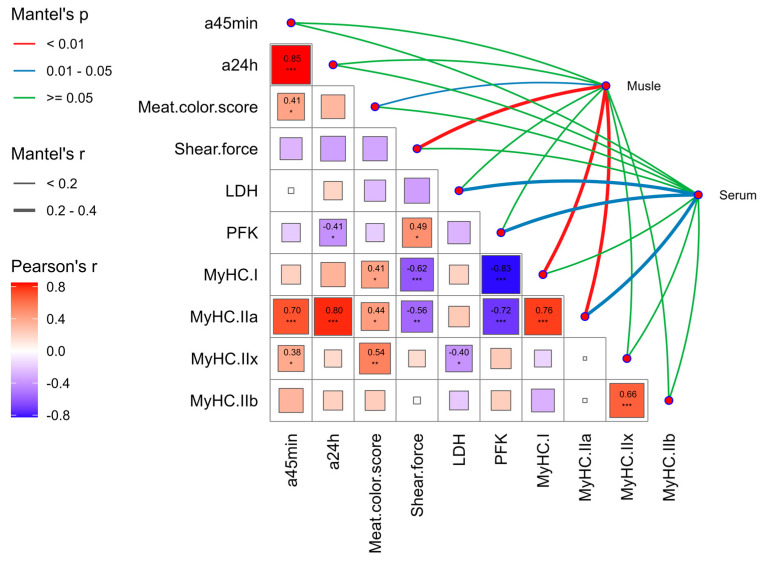
Correlation analysis between antioxidant capacity, meat quality, glycolysis, and muscle fiber type in finishing pigs based on the Mantel-test at 45min; the redness values of the LD muscle at 45 min; the redness values of the LD muscle at 24 h; LDH, Lactate Dehydrogenase; PFK, Phosphofructokinase. Interaction of antioxidant capacity with meat quality, glycolysis, and muscle fiber type in finishing pigs. Significance was expressed as * *p* < 0.05, ** *p* < 0.01, *** *p* < 0.001.

As shown in Figure 3, the antioxidant capacity of the LD muscle was significantly correlated with shear force, *MyHC I* and *MyHC IIa* (Mantel’s *p* < 0.01), as well as with flesh color score (Mantel’s *p* < 0.05). Additionally, serum antioxidant capacity was significantly correlated with lactate dehydrogenase and PFK, and the *MyHC IIa* was also significantly correlated (Mantel’s *p* < 0.05). On the other hand, the redness value of 45 min was positively correlated with the redness value of 24 h, meat color score, *MyHC IIa* and *MyHC IIx* (Pearson’s *p* < 0.05); the redness value of 24 h was negatively correlated with PFK (Pearson’s *p* < 0.05) and positively correlated with *MyHC IIa* (Pearson’s *p* < 0.001); the flesh color score was positively correlated with *MyHC I*, *MyHC IIa*, and *MyHC IIx* (Pearson’s *p* < 0.05); shear force was positively correlated with PEK (Pearson’s *p* < 0.05), negatively correlated with *MyHC I* and *MyHC IIa* (Pearson’s *p* < 0.001); LDH was negatively correlated with *MyHC IIx* (Pearson’s *p* < 0.05); PFK was negatively correlated with *MyHC I* and *MyHC IIa* (Pearson’s *p* < 0.01); *MyHC I* was positively correlated with *MyHC IIa* (Pearson’s *p* < 0.001); *MyHC IIb* and *MyHC IIx* were positively correlated (Pearson’s *p* < 0.001).

## 4. Discussion

This study assessed the effects of FFAI on carcass traits, meat quality, and antioxidant status of finishing pigs. Despite the initial hypothesis that adding FFAI to the diet would impact the growth performance of pigs, the results indicated that while the addition of FFAI did increase the average daily gain and decrease the feed-to-weight ratio to a certain extent, these changes were not statistically significant when compared to the control group. This nonsignificant finding may be attributed to the lipid-lowering effect of FFAI, which consequently led to no notable changes in body weight and feed-to-weight ratio. Generally, too thick backfat thickness and abdominal fat deposition are harmful to pig carcass quality [34]. Our study found that by adding 0.12% FFAI to the diet of finishing pigs, the backfat thickness and perirenal fat percentage can be significantly reduced. This finding is consistent with previous research [20,35], indicating that plant flavonoids have the potential to regulate fat metabolism and improve meat quality. The decrease in backfat thickness and perirenal fat percentage in finishing pigs may be due to the antioxidant effect of FFAI, which can reduce oxidative stress and shield cells from free radical damage, thus reducing fat accumulation. Flavonoids activate the intracellular peroxisome proliferator-activated receptor (PPAR) and related coactivators that promote the oxidation of fatty acids, thus reducing fat accumulation [20].

Pork quality is a key factor that consumers consider when choosing what to buy; not only does it determine the eating experience, but it is also directly related to food safety and health. Therefore, it is crucial to have a thorough assessment of pork quality [2]. In this study, significant improvements in meat quality were observed by adding different concentrations of FFAI to the diet of finishing pigs. Specifically, supplementing the feed with 0.12% FFAI led to a significant enhancement in the redness value and overall meat color of pork, potentially due to an increased antioxidant capacity. Studies have shown that an increase in antioxidant capacity can significantly enhance the redness value and meat color score of pork [36,37]. When ROS produced in the animal body are not removed in a timely manner, an imbalance between oxidation and antioxidation occurs. Antioxidant enzymes, such as SOD, GSH-Px, and CAT, play a pivotal role in mitigating the damage caused by ROS to biological macromolecules [38]. The high antioxidant enzyme activity in muscle indicates that a strong antioxidant system can protect muscle cells from oxidative stress damage, thereby maintaining the structural integrity and functionality of muscle cells [39,40]. In addition, the improvement of antioxidant capacity can also protect the integrity of the cell membrane and reduce the loss of cytoplasm caused by the increase in cell membrane permeability, which is essential for maintaining the water-holding capacity of muscle [41,42]. In this study, it was observed that adding FFAI to the diet resulted in an increase in the total antioxidant capacity in both serum and LD muscle, as well as an elevation in the activities of catalase and glutathione peroxidase in LD muscle. Additionally, it led to a reduction in the malondialdehyde content in both serum and LD muscle. Research indicates that muscle shear force is primarily influenced by muscle fiber type and intramuscular fat. Specifically, when fast-twitch muscle fibers transition into slow-twitch muscle fibers and intramuscular fat increases, muscle shear force decreases, ultimately enhancing muscle tenderness [3]. There exists a positive correlation between drip loss and lactic acid content. As anaerobic glycolysis increases in the muscle, lactic acid accumulates, leading to an increase in drip loss [43]. The effect of glycolysis is determined by glycogen content, which is mainly affected by pre-slaughter activity or stress level, and is also affected by muscle fiber type to a certain extent. The acceleration of the glycolysis rate will reduce the water-holding capacity of muscle [44,45]. In this study, dietary FFAI supplementation decreased the content of pyruvate and lactic acid in the LD muscle of pigs, possibly by inhibiting the rate of pyruvate producing lactic acid to slow down anaerobic glycolysis, thereby reducing drip loss and shear force.

It is well-established that muscle fiber types undergo continuous transformation during growth, and these changes are influenced by both nutritional and non-nutritional factors. These factors exert a significant influence on muscle quality and are intimately linked to various aspects of meat quality, including water-holding capacity, meat color, and tenderness. [2,12]. Some animal experiments have indicated that the addition of plant polyphenols such as apple polyphenols, resveratrol, and proanthocyanidins to the feed can increase the proportion of slow-twitch muscle fibers [46,47,48]. FFAI, which is also a category of plant polyphenols, has been the subject of our research. We have discovered that dietary supplementation with FFAI significantly enhances the expression levels of *MyHC I* (slow-twitch muscle fibers) and *MyHC IIa* (fast-twitch muscle fibers). Moreover, the group supplemented with 0.12% FFAI exhibited significantly higher expression levels of both *MyHC I* and *MyHC IIa* compared with the other two groups. Type I fibers belong to the slow oxidation type, with higher myoglobin content and oxidative metabolism ability, lower glycogen level, and type IIa fibers belong to the fast oxidation type. Their high levels contribute to high redness value, water-holding capacity, and tenderness [49,50]. The type IIx muscle fibers belong to the intermediate muscle fibers, while the type IIb muscle fibers belong to the fast glycolytic muscle fibers [2]. Although they were not significantly different in this study, we subsequently measured that the addition of 0.12% FFAI to the diet can significantly reduce the content of lactic acid, glucose, phosphofructokinase, and pyruvate in LD muscle, indicating that FFAI can slow down the anaerobic glycolysis of pigs after death.

Electronic nose technology is a detection system based on the simulation of the animal olfactory process. It is composed of three systems: gas sensor display, pattern recognition, and information processing [51]. In our study, two main principal components were identified by principal component analysis (PCA): PC1 and PC2. Among them, the contribution rate of PC1 to the total variance reached 60.59%, while the contribution rate of PC2 was 21.49%. The comprehensive contribution rate of these two principal components is 82.08%, more than 80%, indicating that our analysis results can fully reflect the overall characteristics of the three groups of samples. In the resulting figure, the distribution of the experimental groups supplemented with 0.12% and 0.24% FFAI in the feed had a certain degree of overlap, indicating that they were similar in odor characteristics. In addition, there was no overlap between the two experimental groups and the control group, indicating that the addition of FFAI significantly changed the odor characteristics of pork. These results reveal that FFAI concentration has a significant effect on odor characteristics and provides new perspectives to further the role of FFAI in odor formation. Through the electronic nose odor response value analysis, we found that the response values of W1C (sensitive to aromatic components), W5C (sensitive to alkane aromatic components), W5S (sensitive to nitrogen oxides), W3C (sensitive to benzene), and W3S (sensitive to alkanes) in LD muscle of the FFAI group were significantly different from those of the control group. These results indicate that the FFAI group may contain more aromatic substances, nitrogen oxides, and alkanes, which is consistent with the results obtained by GC × GC-MS measurements. In this experiment, the FFAI-added group in the feed showed a significant increase in tenderness, appearance, aroma, and taste in sensory evaluation compared with the control group, which corresponded to the decrease in shear force, the increase in redness values, the increase in meat color scores as well as the enhancement of amino acid composition and flavor substances of pork in the FFAI-added group.

Free amino acids play a central role in pork food science, which are the key factors affecting the sensory quality of food. As the primary taste-active molecules, free amino acids not only directly contribute to the flavor of pork, but also interact with other flavor components to form the overall pork flavor [52]. These amino acids exist in free form in pork, which can stimulate and enhance specific taste sensations, thus enriching the flavor level of pork. Based on their chemical structures and taste characteristics, free amino acids can be categorized into several main taste groups: umami amino acids, sweet amino acids, and bitter amino acids [33]. Serine and threonine can function as sweet-tasting compounds and can also undergo Strecker degradation to produce aldehydes, which subsequently facilitate the formation of pyrazines, compounds that possess a potent aroma [53]. Glutamate is a flavor-enhancing amino acid that imparts a savory taste known as umami, often associated with the freshness and depth of flavor in various foods [54]. Valine and isoleucine are typically categorized as bitter-tasting amino acids, and their elevated concentrations in meat can potentially confer a bitter or otherwise aversive flavor profile [55]. In our study, compared to the control group, supplementing the diet with 0.24% FFAI led to a significant increase in the content of the umami amino acid glutamate and the essential amino acid lysine. However, the levels of the bitter-tasting amino acids, valine and isoleucine, were also notably increased. In contrast, the group supplemented with 0.12% FFAI showed a significant increase in the content of the sweet-tasting amino acids serine and threonine when compared with the other two groups. These results indicate that the incorporation of FFAI not only modulates the expression of specific amino acids but also exerts a potential regulatory effect on the flavor profile of meat products. FFAI may indirectly affect the concentration of free amino acids by regulating muscle metabolism, including glycolysis and antioxidant capacity, and intestinal microbial balance [21,24].

The genesis of pork flavor is a complex process that arises from the intricate interplay of various chemical compounds, including the degradation of flavor precursors (protein degradation, oil degradation, sugar degradation, thiamine degradation), lipid oxidation, Maillard reaction, and the interaction between Maillard reaction products and lipid oxidation products. This process encompasses volatile organic compounds such as aldehydes, esters, alcohols, and aromatic compounds, which contribute significantly to the unique aroma profile of pork [56,57,58]. The objective of the current study is to comprehensively examine the influence of these volatile compounds on the flavor characteristics of pork LD muscle. Aldehydes, which are a category of lipid oxidation and thermal degradation products, are recognized as crucial contributors to the flavor profile of pork products due to their low odor thresholds [59]. Nonanal and hexanal are common volatile compounds found in pork products, primarily generated through the degradation of unsaturated fatty acids [60]. Nonanal, which also has a low odor threshold, contributes a citrus and grassy aroma to the pork’s LD muscle [61]. Like aldehydes, alcohols are also one of the primary products of lipid oxidation, but they possess relatively higher odor thresholds and thus contribute less significantly to the flavor profile compared with aldehydes. It helps to enhance the meaty aroma, endowing the pork with more enticing flavor characteristics. Although esters typically exhibit fruity aromas, they make relatively minor contributions to the overall flavor of meat products because of their high odor thresholds [59]. Among other volatile compounds, benzene exhibits a sweet taste, which contributes to the enhancement of the meat’s flavor profile [57]. In our study, a cluster analysis was conducted on the flavor substances of the longissimus dorsi muscle among three groups, revealing significant differences in the flavor profiles. Compared to the control group, incorporating FFAI into the diet significantly elevated the content of flavor substances, such as 3-pentanone and octanal. Specifically, the inclusion of 0.12% FFAI in the diet notably elevated the levels of 1-penten-3-ol, benzene, and benzaldehyde among the flavor compounds. 1-Penten-3-ol, a volatile compound present in pork, possesses the aroma of cooked meat and has a positive impact on the flavor of pork [62,63]. 3-pentanone is mainly produced by the thermal oxidation or degradation of unsaturated fatty acids and the degradation of amino acids, which may have a synergistic effect on the formation of pig flavor [64]. Benzaldehyde, known for its delightful fruity aroma and low perception threshold, further enriches the flavor profile of pork [11]. These aforementioned flavor substances possess odors characteristic of sweetness (e.g., benzene), and citrus-like scents (e.g., octanal), which may be indicative of the characteristic substances responsible for the enhanced pork flavor in the FFAI-supplemented groups. On the other hand, the group supplemented with 0.24% FFAI exhibited a markedly higher content of ester flavor substances compared with the other two groups. Ester compounds are usually associated with fruit and floral aromas, and their increase may increase the intensity and complexity of pork flavor [65]. These findings may account for the differences observed in both the taste experiments and the electronic nose analyses.

Our study investigated the interplay among the antioxidant capacity of finishing pigs, meat quality, glycolysis, and muscle fiber types. We found that, according to Mantel tests, serum antioxidant capacity was significantly correlated with lactate dehydrogenase, phosphofructokinase, and myosin heavy chain IIa. Additionally, the antioxidant capacity of the LD muscle exhibited a highly significant correlation with shear force, myosin heavy chain IIa, and myosin heavy chain I, as well as a significant correlation with meat color scores [66,67]. These findings indicate that antioxidant capacity plays a significant role in meat quality, the glycolytic process, and muscle fiber typing, particularly in the LD muscle. Moreover, there is a close relationship between meat quality, the glycolytic process, and muscle fiber types. These elements collectively contribute to the formation of meat quality and are crucial for enhancing the quality of pork.

## 5. Conclusions

In summary, the addition of an appropriate amount of FFAI to the diet can reduce the backfat thickness and perirenal fat percentage of finishing pigs, as well as drip loss, water-holding capacity, and shear force, while suppressing anaerobic glycolysis to improve meat color and flavor. Concurrently, it enhances the antioxidant capacity of the pigs, thereby improving meat quality, glycolysis, and muscle fiber types. Mantel test revealed a significant correlation between antioxidant capacity and meat quality, glycolytic processes, and muscle fiber types. In this study, the optimal addition level of FFAI was 0.12%.

## Figures and Tables

**Table 1:** Effect of dietary FFAI supplementation on growth performance of finishing pigs.

Items	FFAI Additions			SEM	*p*-Value
	0	0.12%	0.24%		
Initial weight, kg	73.26	74.27	73.61	0.85	0.90
Final weight, kg	126.59	131.33	130.16	1.46	0.41
ADG, g/d	0.95	1.02	1.01	0.02	0.40
ADFI, kg/d	3.15	3.25	3.15	0.05	0.60
F/G	3.31	3.19	3.14	0.05	0.36

ADG, average daily gain, g/d; ADFI, average daily feed intake, kg/d; F/G, feed-to-gain ratio. Values are the mean ± SEM (*n* = 10).

**Table 2:** Effect of FFAI addition to the diet on carcass traits of finishing pigs.

Items	FFAI Additions			SEM	*p*-Value
	0	0.12%	0.24%		
Average body weight, kg	124.22	126.06	126.20	0.54	0.12
Carcass weight, kg	94.54	96.51	96.43	0.50	0.20
Carcass straight length, cm	101.70	102.67	101.80	0.45	0.65
Carcass slanting length, cm	86.80	88.00	87.50	0.39	0.46
Dressing percentage, %	76.11	76.56	76.41	0.19	0.66
LMA, cm^2^	37.48	42.09	37.67	1.05	0.21
Backfat depth, mm	31.97 ^a^	28.73 ^b^	29.93 ^ab^	0.51	0.03
Perirenal fat weight	1.98	1.76	1.82	0.08	0.57
Perirenal fat percentage, %	2.01 ^a^	1.61 ^b^	1.88 ^a^	0.06	0.01

LMA, Longissimus Muscle Area, cm^2^. Means within a row with different superscript letters are significantly different (*p* < 0.05). Values are the mean ± SEM (*n* = 10).

**Table 3:** Effect of dietary FFAI on quality of fattening pork.

Items	FFAI Additions			SEM	*p*-Value
	0	0.12%	0.24%		
**Post-Slaughter 45 min**					
pH_45 min_	6.20	6.21	6.19	0.04	0.97
L*	45.12	45.4	46.06	0.24	0.26
A*	13.07 ^ab^	13.59 ^a^	12.89 ^b^	0.12	0.04
B*	4.10	4.21	4.55	0.09	0.12
**Post-Slaughter 24 h**					
pH_24 h_	5.66	5.63	5.61	0.02	0.50
L*	51.47	51.37	52.47	0.39	0.46
a*	14.09 ^b^	14.87 ^a^	14.41 ^ab^	0.13	0.03
b*	7.97	8.13	8.65	0.22	0.43
Meat color score	3.41 ^b^	3.69 ^a^	3.33 ^b^	0.06	0.03
Marbling score	1.62	1.63	1.42	0.07	0.38
Drip loss, %	2.9 ^a^	2.09 ^b^	1.64 ^c^	0.12	<0.01
Cooking loss, %	28.21	30.34	28.68	0.39	0.06
Water loss percentage, %	32.40 ^a^	32.30 ^a^	28.23 ^b^	0.75	0.03
Shear force, N	78.86 ^a^	60.57 ^b^	70.99 ^ab^	2.47	<0.01

L*, Lightness; a*, redness value; b*, yellowness value. Means within a row with different superscript letters are significantly different (*p* < 0.05). Values are the mean ± SEM (*n* = 10).

**Table 4:** Effects of dietary FFAI supplementation on sensory indexes of LD muscle in finishing pigs.

Items	FFAI Additions			SEM	*p*-Value
	0	0.12%	0.24%		
Elasticity	5.82	5.86	5.93	0.08	0.88
Juicy	5.34	5.81	5.63	0.10	0.14
Chewiness	5.55	5.66	5.65	0.09	0.88
Tenderness	5.32 ^b^	5.98 ^a^	5.87 ^a^	0.09	<0.01
Shape	6.20 ^b^	6.81 ^a^	6.42 ^b^	0.09	0.01
Flavor	5.45 ^b^	5.74 ^a^	5.79 ^a^	0.06	0.04
Taste	5.30 ^b^	5.80 ^a^	5.74 ^a^	0.08	0.01

Means within a row with different superscript letters are significantly different (*p* < 0.05). Values are the mean ± SEM (*n* = 10).

**Table 5:** Effect of dietary FFAI supplementation on free amino acids in LD muscle of finishing pigs. (Fresh weight basis, μg/g).

Items	FFAI Additions			SEM	*p*-Value
	0	0.12%	0.24%		
**Sweet Amino Acids**					
Ser	43.92 ^b^	52.46 ^a^	43.59 ^b^	1.11	<0.01
Arg	45.91	50.60	47.66	0.85	0.07
Gly	139.59	144.9	141.76	2.08	0.60
Thr	35.06 ^b^	41.64 ^a^	37.27 ^b^	0.80	<0.01
Ala	213.91	211.03	213.69	2.63	0.90
**Fresh Amino Acids**					
Asp	12.07	13.19	12.85	0.52	0.68
Glu	40.87 ^b^	42.73 ^b^	48.97 ^a^	1.10	<0.01
**Bitter Amino Acids**					
His	17.44	17.52	19.13	0.41	0.15
Met	9.87	9.20	10.36	0.29	0.29
Val	37.47 ^b^	36.96 ^b^	42.90 ^a^	0.97	0.01
Ile	27.28 ^b^	26.34 ^b^	31.73 ^a^	0.84	0.01
Leu	49.14	48.10	52.35	1.14	0.30
Phe	30.30	29.49	30.04	0.70	0.90
**Other Amino Acids**					
Pro	22.61	27.66	26.69	1.08	0.12
Cys	4.24	4.23	3.50	0.21	0.25
Lys	34.00 ^b^	33.86 ^a^	37.29 ^a^	0.66	0.05
Tyr	24.74	24.88	25.60	0.52	0.47
Trp	5.14	4.34	4.81	0.17	0.16

Asp, aspartic acid; Glu, glutamic acid; His, histidine; Ser, serine; Arg, arginine; Gly, glycine; Thr, threonine; Ala, alanine; Pro, proline; Cys, cysteine; Lys, lysine; Tyr, tyrosine; Met, methionine; Val, valine; Ile, isoleucine; Leu, leucine; Phe, phenylalanine; Trp, tryptophan; Means within a row with different superscript letters are significantly different (*p* < 0.05). Values are the mean ± SEM (*n* = 10).

## Data Availability

The data supporting the conclusion of this article is included within the article and Supplementary Materials.

## Supplementary Materials

The following supporting information can be downloaded at: https://www.mdpi.com/article/10.3390/antiox13111385/s1, Table S1. Composition of FFAI.; Table S2. Composition and nutrient levels of the basal diets; Table S3. Sensory evaluation scoring rules; Table S4. The odor type represented by the sensor; Table S5. Gradient elution procedures; Table S6. Primers used for quantitative real-time PCR.

## References

[1] Dunshea F.R., Pluske J.R., Ponnampalam E.N. (2024). Dietary iron or inulin supplementation alters iron status, growth performance, intramuscular fat and meat quality in finisher pigs. Meat Sci..

[2] Li Y., Feng Y., Chen X., He J., Luo Y., Yu B., Chen D., Huang Z. (2024). Dietary short-term supplementation of grape seed proanthocyanidin extract improves pork quality and promotes skeletal muscle fiber type conversion in finishing pigs. Meat Sci..

[3] Chen J., Li J., Liu X., He Y. (2021). Effects of dietary fat saturation level on growth performance, carcass traits, blood lipid parameters, tissue fatty acid composition and meat quality of finishing pigs. Anim. Biosci..

[4] Duffy S.K., Kelly A.K., Rajauria G., Jakobsen J., Clarke L.C., Monahan F.J., Dowling K.G., Hull G., Galvin K., Cashman K.D. (2018). The use of synthetic and natural vitamin D sources in pig diets to improve meat quality and vitamin D content. Meat Sci..

[5] Deng L., Hao S., Zou W., Wei P., Sun W., Wu H., Lu W., He Y. (2023). Effects of Supplementing Growing-Finishing Crossbred Pigs with Glycerin, Vitamin C and Niacinamide on Carcass Characteristics and Meat Quality. Animals.

[6] Remole H.M., Htoo J.K., Mendoza S.M., Bradley C.L., Dilger R.N., Dilger A.C., Harsh B.N. (2024). Effects of supplemental methionine sources in finishing pig diets on growth performance, carcass characteristics, cutting yields, and meat quality. Transl. Anim. Sci..

[7] Park T.W., Lee E.Y., Jung Y., Son Y.M., Oh S.H., Kim D.H., Lee C.Y., Joo S.T., Jang J.C. (2023). Effects of lysine concentration of the diet on growth performance and meat quality in finishing pigs with high slaughter weights. J. Anim. Sci. Technol..

[8] Scerra M., Foti F., Caparra P., Cilione C., Bognanno M., Paolo F., Paolo C., Natalello A., Musati M., Chies L. (2024). Effects of feeding bergamot pulp and olive leaves on performance and meat quality in Apulo-Calabrese pigs. Vet. Anim. Sci..

[9] Xu X., Chen X., Chen D., Yu B., Yin J., Huang Z. (2019). Effects of dietary apple polyphenol supplementation on carcass traits, meat quality, muscle amino acid and fatty acid composition in finishing pigs. Food Funct..

[10] Wu W., Zhan J., Tang X., Li T., Duan S. (2022). Characterization and identification of pork flavor compounds and their precursors in Chinese indigenous pig breeds by volatile profiling and multivariate analysis. Food Chem..

[11] Bi J., Li Y., Yang Z., Lin Z., Chen F., Liu S., Li C. (2022). Effect of different cooking times on the fat flavor compounds of pork belly. J. Food Biochem..

[12] Zhang C., Luo J., Yu B., Zheng P., Huang Z., Mao X., He J., Yu J., Chen J., Chen D. (2015). Dietary resveratrol supplementation improves meat quality of finishing pigs through changing muscle fiber characteristics and antioxidative status. Meat Sci..

[13] Zhang X., Zhang Z., Sun Y., Liu Y., Zhong X., Zhu J., Yu X., Lu Y., Lu Z., Sun X. (2023). Antioxidant Capacity, Inflammatory Response, Carcass Characteristics and Meat Quality of Hu Sheep in Response to Dietary Soluble Protein Levels with Decreased Crude Protein Content. Antioxidants.

[14] Rossi R., Pastorelli G., Cannata S., Tavaniello S., Maiorano G., Corino C. (2013). Effect of long term dietary supplementation with plant extract on carcass characteristics meat quality and oxidative stability in pork. Meat Sci..

[15] Soares M.H., Júnior D.T.V., de Amorim Rodrigues G., Júnior R.L.C., Rocha G.C., Bohrer B.M., Juárez M., de Souza Duarte M., Saraiva A. (2022). Effects of feeding ractopamine hydrochloride with or without supplemental betaine on live performance, carcass and meat quality traits, and gene expression of finishing pigs. Meat Sci..

[16] Zhu C.-Q., Chen J.-B., Zhao C.-N., Liu X.-J., Chen Y.-Y., Liang J.-J., Cao J.-P., Wang Y., Sun C.-D. (2023). Advances in extraction and purification of citrus flavonoids. Food Front..

[17] Bollikolla H.B., Tyagi R., Gokada M.R., Anandam R., Kasthuri J.K., Durga V.T., Alam M.M., Mannam K.M. (2022). Flavones as Important Scaffolds for Anticancer, Antioxidant and Anti-Tubercular Activities: An Overview of Reports 2015-2020. Mosc. Univ. Chem. Bull..

[18] Abualhasan M., Jaradat N., Al-Rimawi F., Shahwan M., Mansour D., Alhend Z., Alsoroghli Y., Mousa A. (2022). Bioactivity evaluation of synthesized flavone analogs. Food Sci. Technol..

[19] Agrawal K.K., Murti Y. (2022). Tangeretin: A Biologically Potential Citrus Flavone. Curr. Tradit. Med..

[20] Alam F., Mohammadin K., Shafique Z., Amjad S.T., bin Asad M.H.H. (2022). Citrus flavonoids as potential therapeutic agents: A review. Phytother. Res..

[21] Baky M.H., Elshahed M., Wessjohann L., Farag M.A. (2022). Interactions between dietary flavonoids and the gut microbiome: A comprehensive review. Br. J. Nutr..

[22] Mahfuz S., Mun H.S., Dilawar M.A., Ampode K.M.B., Yang C.J. (2022). Potential Role of Protocatechuic Acid as Natural Feed Additives in Farm Animal Production. Animals.

[23] Wang Q., Zhang N., Cui K., Wang S., Lyu X., Diao Q. (2018). Effects of Lactobacillus Plantarum, Buckwheat Flavone and Their Compounds on Growth Performance, Nutrient Digestibility and Serum Indices of Weaned Piglets. Chin. J. Anim. Nutr..

[24] Cui K., Wang Q., Wang S., Diao Q., Zhang N. (2019). The Facilitating Effect of Tartary Buckwheat Flavonoids and *Lactobacillus plantarum* on the Growth Performance, Nutrient Digestibility, Antioxidant Capacity, and Fecal Microbiota of Weaned Piglets. Animals.

[25] Yuan D., Tan B.E., Zha A., Liao P. (2022). Interventional Effect of *Eucommia ulmoides* Flavones on Growth Inhibition, Oxidative Stress Injury and Inflammatory Injury of Weaned Piglets Induced by Deoxynivalenol. Chin. J. Anim. Nutr..

[26] Chen B., Luo J., Han Y., Du H., Liu J., He W., Zhu J., Xiao J., Wang J., Cao Y. (2021). Dietary Tangeretin Alleviated Dextran Sulfate Sodium-Induced Colitis in Mice via Inhibiting Inflammatory Response, Restoring Intestinal Barrier Function, and Modulating Gut Microbiota. J. Agric. Food Chem..

[27] Li H.-L., Wei Y.-Y., Li X.-H., Zhang S.-S., Zhang R.-T., Li J.-H., Ma B.-W., Shao S.-B., Lv Z.-W., Ruan H. (2022). Diosmetin has therapeutic efficacy in colitis regulating gut microbiota, inflammation, and oxidative stress via the circ-Sirt1/Sirt1 axis. Acta Pharmacol. Sin..

[28] Vajdi M., Farhangi M.A. (2023). Citrus peel derived poly-methoxylated flavones (PMF): A systematic review of the potential bioactive agents against obesity and obesity related metabolic disorders. Int. J. Vitam. Nutr. Res..

[29] Tian Z., Cui Y., Lu H., Wang G., Ma X. (2021). Effect of long-term dietary probiotic Lactobacillus reuteri 1 or antibiotics on meat quality, muscular amino acids and fatty acids in pigs. Meat Sci..

[30] Konarska M., Kuchida K., Tarr G., Polkinghorne R.J. (2017). Relationships between marbling measures across principal muscles. Meat Sci..

[31] Zhang H., Zheng Y., Zha X., Liu X., Ma Y., Loor J.J., Elsabagh M., Wang M., Wang H., Jiang H. (2022). Dietary N-carbamylglutamate and L-arginine supplementation improves redox status and suppresses apoptosis in the colon of intrauterine growth-retarded suckling lambs. Anim. Nutr..

[32] Sui Y., Harvey P.J. (2021). Effect of Light Intensity and Wavelength on Biomass Growth and Protein and Amino Acid Composition of *Dunaliella salina*. Foods.

[33] Xu D., Wang Y., Zhang X., Yan E., He L., Wang L., Ma C., Zhang P., Yin J. (2022). Dietary Valine/Isoleucine Ratio Impact Carcass Characteristics, Meat Edible Quality and Nutritional Values in Finishing Crossbred Duroc × Landrace × Yorkshire Pigs with Different Slaughter Weights. Front. Nutr..

[34] Li Y., Liu Y., Li F., Lin Q., Dai Q., Sun J., Huang X., Chen X., Yin Y. (2018). Effects of dietary ramie powder at various levels on carcass traits and meat quality in finishing pigs. Meat Sci..

[35] Kariagina A., Doseff A.I. (2022). Anti-Inflammatory Mechanisms of Dietary Flavones: Tapping into Nature to Control Chronic Inflammation in Obesity and Cancer. Int. J. Mol. Sci..

[36] Feng Y., Chen X., Chen D., He J., Zheng P., Luo Y., Yu B., Huang Z. (2023). Dietary grape seed proanthocyanidin extract supplementation improves antioxidant capacity and lipid metabolism in finishing pigs. Anim. Biotechnol..

[37] Wang T., Li J., Shao Y., Yao W., Xia J., He Q., Huang F. (2020). The effect of dietary garcinol supplementation on oxidative stability, muscle postmortem glycolysis and meat quality in pigs. Meat Sci..

[38] Huang Q., Xu W., Bai K.W., He J.T., Ahmad H., Zhou L., Zhang L.L., Wang T. (2017). Protective effects of leucine on redox status and mitochondrial-related gene abundance in the jejunum of intrauterine growth-retarded piglets during early weaning period. Arch. Anim. Nutr..

[39] Zhang P., Zhang H., Ma C., Lv Q., Yu H., Zhang Q. (2024). Effect of ginseng stem leaf extract on the production performance, meat quality, antioxidant status, immune function, and lipid metabolism of broilers. Front. Vet. Sci..

[40] Zhang L., Yang D., Luo R., Luo Y., Hou Y. (2024). Research Progress on the Mechanism of the Impact of Myofibrillar Protein Oxidation on the Flavor of Meat Products. Foods.

[41] Zhang G., Huang J., Sun Z., Guo Y., Lin G., Zhang Z., Zhao J. (2024). Effects of Trace Mineral Source on Growth Performance, Antioxidant Activity, and Meat Quality of Pigs Fed an Oxidized Soy Oil Supplemented Diet. Antioxidants.

[42] Weng G., Yu M., Deng C., Liu Y., Song M., Deng J., Yin Y., Ma X., Deng D. (2024). Effects of dietary Brevibacillus laterosporus BL1 supplementation on meat quality, antioxidant capacity, and the profiles of muscle amino acids and fatty acids in finishing pigs. Meat Sci..

[43] Wang Y., Zhang H., Yan E., He L., Guo J., Zhang X., Yin J. (2023). Carcass and meat quality traits and their relationships in Duroc × Landrace × Yorkshire barrows slaughtered at various seasons. Meat Sci..

[44] Zhang Z.Y., Jia G.Q., Zuo J.J., Zhang Y., Lei J., Ren L., Feng D.Y. (2012). Effects of constant and cyclic heat stress on muscle metabolism and meat quality of broiler breast fillet and thigh meat. Poult. Sci..

[45] Apaoblaza A., Galaz A., Strobel P., Ramírez-Reveco A., Jeréz-Timaure N., Gallo C. (2015). Glycolytic potential and activity of adenosine monophosphate kinase (AMPK), glycogen phosphorylase (GP) and glycogen debranching enzyme (GDE) in steer carcasses with normal (<5.8) or high (>5.9) 24 h pH determined in M. longissimus dorsi. Meat Sci..

[46] Mizunoya W., Okamoto S., Miyahara H., Akahoshi M., Suzuki T., Do M.Q., Ohtsubo H., Komiya Y., Qahar M., Waga T. (2017). Fast-to-slow shift of muscle fiber-type composition by dietary apple polyphenols in rats: Impact of the low-dose supplementation. Anim. Sci. J..

[47] Xu M., Chen X., Huang Z., Chen D., Li M., He J., Chen H., Zheng P., Yu J., Luo Y. (2022). Effects of dietary grape seed proanthocyanidin extract supplementation on meat quality, muscle fiber characteristics and antioxidant capacity of finishing pigs. Food Chem..

[48] Li J., Liang R., Mao Y., Yang X., Luo X., Qian Z., Zhang Y., Zhu L. (2022). Effect of dietary resveratrol supplementation on muscle fiber types and meat quality in beef cattle. Meat Sci..

[49] Ying F., Zhang L., Bu G., Xiong Y., Zuo B. (2016). Muscle fiber-type conversion in the transgenic pigs with overexpression of PGC1α gene in muscle. Biochem. Biophys. Res. Commun..

[50] Kim G.D., Jeong J.Y., Jung E.Y., Yang H.S., Lim H.T., Joo S.T. (2013). The influence of fiber size distribution of type IIB on carcass traits and meat quality in pigs. Meat Sci..

[51] Han D., Zhang C.H., Fauconnier M.L., Mi S. (2020). Characterization and differentiation of boiled pork from Tibetan, Sanmenxia and Duroc × (Landrac × Yorkshire) pigs by volatiles profiling and chemometrics analysis. Food Res. Int..

[52] Hwang Y.H., Sabikun N., Ismail I., Joo S.T. (2019). Changes in Sensory Compounds During Dry Aging of Pork Cuts. Food Sci. Anim. Resour..

[53] Zhang J., Zhang Y., Wang Y., Xing L., Zhang W. (2020). Influences of ultrasonic-assisted frying on the flavor characteristics of fried meatballs. Innov. Food Sci. Emerg. Technol..

[54] Wang J., Lu R., Li Y., Lu J., Liang Q., Zheng Z., Huang H., Deng F., Huang H., Jiang H. (2023). Dietary supplementation with jasmine flower residue improves meat quality and flavor of goat. Front. Nutr..

[55] Wang D., Chen G., Chai M., Shi C., Geng Y., Che Y., Li Y., Liu S., Gao Y., Hou H. (2022). Effects of dietary protein levels on production performance, meat quality and flavor of fattening pigs. Front. Nutr..

[56] Aaslyng M.D., Meinert L. (2017). Meat flavour in pork and beef—From animal to meal. Meat Sci..

[57] Sun A., Wu W., Soladoye O.P., Aluko R.E., Bak K.H., Fu Y., Zhang Y. (2022). Maillard reaction of food-derived peptides as a potential route to generate meat flavor compounds: A review. Food Res. Int..

[58] Tu T., Wu W., Tang X., Ge Q., Zhan J. (2021). Screening out important substances for distinguishing Chinese indigenous pork and hybrid pork and identifying different pork muscles by analyzing the fatty acid and nucleotide contents. Food Chem..

[59] Zhao J., Wang M., Xie J., Zhao M., Hou L., Liang J., Wang S., Cheng J. (2017). Volatile flavor constituents in the pork broth of black-pig. Food Chem..

[60] Saldaña E., Saldarriaga L., Cabrera J., Siche R., Behrens J.H., Selani M.M., de Almeida M.A., Silva L.D., Silva Pinto J.S., Contreras-Castillo C.J. (2019). Relationship between volatile compounds and consumer-based sensory characteristics of bacon smoked with different Brazilian woods. Food Res. Int..

[61] Benet I., Guàrdia M.D., Ibañez C., Solà J., Arnau J., Roura E. (2016). Low intramuscular fat (but high in PUFA) content in cooked cured pork ham decreased Maillard reaction volatiles and pleasing aroma attributes. Food Chem..

[62] Gardner K., Legako J.F. (2018). Volatile flavor compounds vary by beef product type and degree of doneness. J. Anim. Sci..

[63] Li P., Zhou H., Wang Z., Al-Dalali S., Nie W., Xu F., Li C., Li P., Cai K., Xu B. (2022). Analysis of flavor formation during the production of Jinhua dry-cured ham using headspace-gas chromatography-ion mobility spectrometry (HS-GC-IMS). Meat Sci..

[64] Huang G.L., Liu T.T., Mao X.M., Quan X.Y., Sui S.Y., Ma J.J., Sun L.X., Li H.C., Shao Q.S., Wang Y.N. (2023). Insights into the volatile flavor and quality profiles of loquat (*Eriobotrya japonica* Lindl.) during shelf-life via HS-GC-IMS, E-nose, and E-tongue. Food Chem. X.

[65] Wang X., Wang X., Zhang X., Liu S., Yu J., Cui H., Xia S., Ho C.T. (2023). Changes of lipid oxidation, volatile and taste-active compounds during pan-heating of pork belly. Food Res. Int..

[66] Park J., Moon S.S., Song S., Cheng H., Im C., Du L., Kim G.D. (2024). Comparative review of muscle fiber characteristics between porcine skeletal muscles. J. Anim. Sci. Technol..

[67] Lefaucheur L., Edom F., Ecolan P., Butler-Browne G.S. (1995). Pattern of muscle fiber type formation in the pig. Dev. Dyn..

