# Supplementary material for: Effects of Flavonoids in Fructus Aurantii Immaturus on Carcass Traits, Meat Quality and Antioxidant Capacity in Finishing Pigs
Source: Antioxidants (Basel). 2024 Nov 13;13(11):1385. doi: 10.3390/antiox13111385 (PMC11591327; doi:10.3390/antiox13111385)
Supplement: Supplementary file 1 [file antioxidants-13-01385-s001.zip › antioxidants-3253020-supplementary.pdf]

Table S1. Composition of FFAI.

Ingredient	Content	Units
Bioflavonoids	33.11	%
Polysaccharides	9.75	%
Polyphenols	6.51	%
Dietary fiber	33.01	%
Residue on ignition	23.76	%
Synephrine	1.01	%
Hesperidin	11.30	%

The components of flavonoids in Fructus Aurantii Immaturus were detected by ultra-high speed liquid chromatograph.

Table S2. Composition and nutrient levels of the basal diets

Composition	70-90 kg	90-125 kg
**Ingredient(%)**		
Corn	34.40	35.00
Wheat	40.00	45.00
Soybean meal (46) [a]	9.70	3.00
Wheat bran	7.00	8.00
Soybean oil	0.50	0.60
NaCl	0.40	0.40
Fermented material F-100	4.00	4.00
Premix[b]	4.00	4.00
Total	100.00	100.00
**Nutritional levels**[c]		
Digestibility energy (MJ/kg)	3.25	3.24
CP	13.94	11.86
Lys	0.90	0.79
Met	0.25	0.24
Met+Cys	0.51	0.47
Thr	0.59	0.51
Ca	0.62	0.51
Total phosphorus	0.47	0.41
Available phosphorus	0.31	0.24

[a] The protein content of the soybean meal is 46%.

[b]The premix provided the following per kg of diets: 70 to 125 kg stage, VA 6000 IU, VD_3 3000 IU, VE 40 IU, VK_3 3 mg, VB_1 1.8 mg, VB_2 6 mg, VB_6 6 mg, VB_{12} 0.024 mg, Biotin 4.5 mg, Folic acid 0.3 mg, Niacin 24 mg, Pantothenic acid 20 mg, Choline 500 mg, Cu ($CuSO_4 \cdot 5H_2O$) 15 mg, Fe ($FeSO_4$) 100 mg, Mn ($MnSO_4 \cdot H_2O$) 100 mg, Zn ($ZnSO_4$) 50 mg, I (KI) 0.5 mg, Se (Na_2SeO_3) 0.3 mg.

[c] CP, Ca, and P are the measured values, and the rest are the calculated values.

CP, crude protein; Lys, lysine; Met, methionine; Met + Cys, methionine + cysteine; Thr, threonine; Ca, calcium.

Table S3. Sensory evaluation scoring rules

Evaluating indicator	Grading
Elasticity	Pressing rebound quickly (10) .<br>Pressing rebound in general .<br>Inelastic (1).
Juicy	Juicy and full entrance (10)<br>More juice, the entrance is fuller .<br>More juice, general entrance .<br>Less juice, general entrance .<br>Too little juice, dry entrance (1).
Chewiness	The taste is delicate and chewy (10).<br>The taste is slightly hard, chewy in general.<br>Hard taste, poor chewiness (10).
Tenderness	Soft texture, easy to swallow (10).<br>Soft texture, smooth swallowing.<br>Soft and hard moderate.<br>Rough texture, difficult to chew.<br>Hard texture, hard to swallow (10).
Shape	Complete shape (10).<br>The shape is relatively loose.<br>Loose (1).
Flavor	It has a mellow taste and cooked meat aroma (10).<br>Meaty, light smell.<br>No meat flavor, no obvious odor (1).
Taste	Taste full thick (10).<br>Meaty, light in taste.<br>No meat, taste general (1).

Table S4. The odor type represented by the sensor

Serial number	Sensor	Odor type
1	W1C	Aromatic
2	W5S	Broadrange
3	W3C	Aromatic
4	W6S	Hydrogen
5	W5C	Arom-aliph
6	W1S	Broad-methane
7	W1W	Sulphur-organic
8	W2S	Broad-alcohol
9	W2W	Sulph-chlor
10	W3S	Methane-aliph

Table S5. Gradient elution procedures

**Time (min)**	%A	%B	%C	%D	**Curve**
**Initial**	10.0	0.0	90.0	0.0	**Initial**
0.29	9.9	0.0	90.1	0.0	11
5.49	9.0	80.0	11.C	0.0	7
7.10	8.0	15.6	57.9	18.5	6
7.30	8.0	15.6	57.9	18.5	6
7.69	7.8	0.0	70.9	21.3	6
7.99	4.0	0.0	36.3	59.7	6
8.59	4.0	0.0	36.3	59.7	6
8.68	10.0	0.0	90.0	0.0	6
10.20	10.0	0.0	90.0	0.0	6

Table S6. Primers used for quantitative real-time PCR

Genes	Primer sequences(5'-3')	Product size, bp	GenBank
MyHC I	F:GGCCCCTTCCAGCTTGA<br>R:TGGCTGCGCCTTGGTTT	144	XM_006715781.3
MyHC IIa	F:TTAAAAAGCTCCAAGAACTGTTTCA<br>R:CCATTTCCTGGTCGGAACTC	136	NM_022347.5
MyHC IIx	F:AGCTTCAAGTTCTGCCCCACT<br>R:GGCTGCGGGTTATTGATGG	76	XM_017024675.2
MyHC IIb	F:CACTTTAAGTAGTTGTCTGCCTTGAG<br>R:GGCAGCAGGGCACTAGATGT	80	NM_001349933.1
